# A Multisource Approach to Improving Epidemiologic Estimates: Application to Global B-Cell Malignancies

**DOI:** 10.5402/2012/129713

**Published:** 2012-12-31

**Authors:** Meghan E. Mitchell, Kimberly Lowe, Jon Fryzek

**Affiliations:** ^1^Exponent, Health Sciences Practice, New York, NY 10170, USA; ^2^Exponent, Health Sciences Practice, Alexandria, VA 22314, USA; ^3^Exponent, Health Sciences Practice, Bellevue, WA 98007, USA

## Abstract

The compilation of comprehensive, worldwide epidemiologic data can inform hypotheses on cancer etiology and guide future drug development. These statistics are reported by a multitude of sources using varying methods; thus, compiling a complete database of these statistics is a challenge. To this end, this paper examined the usefulness of a novel, multisource approach—extracting data from the peer-reviewed literature, online reports, and query systems from cancer registries and health agencies and directly contacting cancer registry personnel—for building a comprehensive, multinational epidemiologic cancer database. The major B-cell malignancies were chosen as the cancer subtype to test this approach largely because their epidemiology has not been well characterized in the peer-reviewed literature. We found that a multisource approach yields a more comprehensive epidemiologic database than what would have been possible with the use of literature searches alone. In addition, our paper revealed that cancer registries vary considerably in their methodology, comprehensiveness, and ability to gather information on specific B-cell malignancy subtypes. Collectively, this paper demonstrates the feasibility and value of a multisource approach to gathering epidemiologic data.

## 1. Introduction

Descriptive epidemiologic statistics assist public health planning and provide valuable information about the burden of illness to policy makers, funding agencies, resource planners, healthcare insurers, and manufacturers. Information on malignancies that is compatible with their clinical classifications is of particular value to clinicians and public health professionals and increasing efforts are being made to collect data at this detailed level (e.g., the HAEMACARE project [1]). It is challenging, however, to assemble a database of descriptive epidemiologic statistics from the peer-reviewed literature alone. Cancer registries are used worldwide to collect and analyze demographic, diagnostic, and survival data. Some registries are fraught with poor quality and infrastructure; however, there is no standardized system for the collection and reporting of descriptive statistics worldwide [2, 3]. Comprehensive reviews of descriptive epidemiologic statistics, such as this one, are warranted to better understand the totality of the currently available data and to optimize the utility of such data in the future. 

This paper uses a particular cancer subtype—B-cell malignancies—to evaluate a novel approach to assembling a database of worldwide, national-level, descriptive epidemiologic cancer statistics. This approach incorporates information from various sources, including the peer-reviewed literature, online reports, and query systems from cancer registries and health agencies, and direct contact with cancer registries, to provide a current, comprehensive database for a representative group of countries worldwide. The major B-cell malignancies were chosen as the cancer subtype to test this approach largely because their epidemiology has not been well characterized. Further, some B-cell malignancy subtypes require detailed diagnostic evaluation, and this paper allowed us to broadly assess the extent to which detailed diagnoses are currently being reported to cancer registries. This is important because an understanding of these subtypes may inform the development of novel treatments. 

B-cell malignancies emerge in cells of the bone marrow, blood, or other tissues at various stages of B-lymphocyte differentiation and represent a rare (3% of all malignancies) and heterogeneous group of lymphohematopoietic malignancies (Table 1) [4]. In 2001 [5] and 2008 [6], the World Health Organization (WHO) classified lymphoid hematologic neoplasms into 4 major categories based on the cell linage of the malignancy or the normal cell type that the tumor most resembles: B-cell malignancies, T-cell malignancies, natural killer (NK) cell malignancies, and Hodgkin's disease. Further divisions were based on cell maturity (e.g., precursor versus mature B-cell neoplasms), as well as morphologic, genotypic, genetic, immunohistochemical, and clinical criteria. The major B-cell lymphoid malignancies include diffuse large B-cell lymphomas (DLBCLs), follicular lymphomas (FLs), and plasma cell or multiple myeloma (MM). It is estimated that 85% to 90% of non-Hodgkin's lymphomas (NHL) are of B-cell origin, including DLBCL and FL [7]. Certain leukemias also arise from the B cells and are categorized based on (1) whether the disease is acute or chronic and (2) what type of cell is infected. The leukemias associated with abnormalities of B-cell formation in the blood include B-cell acute lymphoblastic leukemia (ALL) and chronic lymphocytic leukemia (CLL) or small lymphocytic lymphoma. Approximately 80% of all ALL cases are of B-cell linage [8]. ALL represents one of the most common forms of leukemia among children. 

In this paper, a novel, comprehensive approach to gathering descriptive, epidemiologic statistics was used to develop a current worldwide database of the major B-cell malignancies. The intent of this paper was to evaluate the feasibility of this novel approach for assembling a database of descriptive statistics and to broadly assess the level of detail collected by cancer registries worldwide. We also briefly describe our findings of the descriptive epidemiology of B-cell malignancies.

## 2. Methods

### 2.1. Data Identification

We collected the most recent information available on the incidence, prevalence, and survival of the following B-cell malignancies: DLBCL, FL, CLL, MM, adult B-cell ALL, and pediatric B-cell ALL. These data were collected for a representative selection of countries worldwide, categorized into the following groups: North and South America (Brazil, Canada, and the United States), the European Union-5 (France, Germany, Italy, Spain, and the United Kingdom), Asia (China, India, Japan, and South Korea), and Australia. Descriptive statistics for the countries constituting the United Kingdom (England, Scotland, Wales, and Ireland [including Northern Ireland and the Republic of Ireland]) were also collected.

We employed three general strategies: a structured literature review of the published, peer-reviewed literature in PubMed, a review of online documentation from cancer registries and relevant health agencies, and, finally, direct contact via email with personnel at cancer registries and key experts in the field. The first 2 strategies were employed in tandem; direct contact was used to address any remaining gaps and clarify the reasons behind missing data.

Various search strings (See Table S1 in Supplementary Material available online at doi:10.5402/2012/129713) were used in the PubMed search to identify general articles describing the epidemiology of these malignancies in the countries of interest. The search was restricted to human studies published from January 1, 2000 to July 13, 2011. Approximately 5600 citations were retrieved, and the titles and abstracts of these publications were reviewed. Studies that appeared to report descriptive epidemiologic statistics (i.e., incidence, prevalence, or survival) for the B-cell malignancies of interest were included. Articles reporting national-level data on the statistics, malignancies, and countries of interest were identified and the relevant information was extracted. All other studies were excluded. In addition, case reports, case series, and letters to the editor were excluded. Relevant full-text articles were retrieved and reviewed for inclusion.

Worldwide, national, and regional cancer registries and online documentation for these registries (including reports and online query systems) were then searched.  Table 2 lists the cancer registries searched by country. The websites of other relevant health agencies and government departments were also searched for reports and references to the data of interest. Registries and relevant agencies were identified from previous experience, online research into the structure of public health in each country, and involvement in organizations such as the International Association of Cancer Registries,[9] GLOBOCAN,[10] and the European Network of Cancer Registries [11]. For some non-English language speaking countries (France, Germany, Spain, China, and Japan), persons proficient in the language searched online data and translated some of the published literature; a translation of non-English language material (including online documentation and published studies) was not performed, however.

The material identified in the PubMed search and online data were compared. The goal was to identify the most recent estimates of the incidence, prevalence, and survival associated with the relevant B-cell malignancies. If relevant data were reported by more than 1 source, the source with the most recent data and/or the most relevant data (e.g., data specific to B-cell ALL versus ALL) was used. Once gaps in the availability of the data of interest were identified, cancer registry personnel and key experts in the field were contacted via e-mail to discuss the availability of missing statistics (these contacts are referred to as “personal communication”).  Table 2 describes the cancer registries and organizations that were contacted and the outcome of each respective communication.

We did not collect data from GLOBOCAN or the Cancer Incidence in Five Continents (CI5) [12] series. These IARC programs provide a valuable resource for modeling estimates on the incidence and mortality of common cancers, including NHL and MM. However, since modeling estimates may not reflect the most recent national-level statistics, we did not rely on these databases for NHL and MM data. There are also standardized efforts in Europe to report descriptive epidemiologic data: EUROCARE [13] and the Automated Childhood Cancer Information System [14]. Similar to CI5; however, these standardized systems are not based on complete ascertainment from registries in the underlying countries, for example, only select regional registries participate in Spain, Italy, and France. As such, we only used data from these European programs in the absence of national-level data.

### 2.2. Data Extraction and Reporting

The following information was extracted from each data source: source of the data, country or countries, malignancy type, including any available information on *International Classification of Diseases* (ICD) codes, and data relevant to incidence, prevalence, and/or survival. We sought to assess the availability of *recent* statistics; therefore, only post-2000 diagnoses were assessed for incidence and survival, and only prevalence estimates for a date after 2000 were collected. Incidence projections for future years were collected only in the absence of actual incidence estimates. Limited-duration prevalence rates and prevalence proportions estimated on a date after 2000 were collected for all available time frames (e.g., 5 y, 10 y). Relative survival rates were collected for all time periods (e.g., 5 y survival, 10 y survival) for diagnoses post-2000; it was noted whether survival rates were estimated using period methods. Relative survival is defined as the ratio of the proportion of observed survivors in a cohort of cancer patients to the proportion of expected survivors in a comparable set of cancer-free individuals. In the absence of data on relative survival, observed survival data were collected. 

Data were collected by gender and methods for age standardization and any age restrictions were noted. Data were reported from multiple citations for a particular malignancy and country if different information sources reported supplementary information.

## 3. Results and Discussion

### 3.1. General Availability of Data from Cancer Registries

Large gaps in the availability of national-level statistics on B-cell malignancies were observed at the outset of our review process and few descriptive statistics were available for the B-cell NHLs (i.e., DLBCL and FL) and B-cell ALL. We, therefore, extended the review to the larger diagnostic categories of NHL and ALL. Data on diffuse NHL, a diagnostic category of NHL that includes DLBCL [6], were available from numerous countries, and data were collected for this larger diagnostic category in the absence of data specific to DLBCL. In addition, few descriptive statistics were available for ALL and CLL. As a result, we collected data on the larger diagnostic category “lymphoid leukemia.” Finally, national-level statistics were lacking in several countries (e.g., Brazil, India, and China) because of the status of cancer registration in these countries. To compensate for this deficiency, data from regional registries were collected in the absence of national estimates. 

Table 3 provides an overview of the availability of statistics by malignancy type and country; notations are provided where proxy data (as defined above) were used. Supplementary Tables 2 to 7 provide the most recent statistics by country and statistic type for NHL, DLBCL and FL, adult ALL, pediatric ALL, MM, and CLL, respectively.

This paper provides insights into the status of cancer registration worldwide. Cancer registries are well recognized as a valuable information source for public health planning and epidemiologic and therapeutic research because they provide essential epidemiologic data on the current burden of disease. Nonetheless, we found that the implementation of cancer registries has been slow in some countries, and data were not made readily available or updated in a timely manner in others. In 2006, almost 80% of the world's population was not covered by a population-based cancer registry; most of this unrepresented population was from low- and middle-income countries [15]. The uncoordinated nature of cancer registration worldwide has resulted in wide variation in the geographical areas covered, the methods used to estimate descriptive statistics, and the level of detail collected. This differential evolution of cancer registration worldwide yielded notable between-country variation in the availability of descriptive statistics on B-cell malignancies (Table 3). The use of online resources and direct contact with cancer registries greatly expanded our database.

The US Surveillance, Epidemiology, and End Results (SEER) registry [16] provided the most complete data on B-cell malignancies. The SEER registry provides incidence, prevalence, and survival data by ICD-O-3 code using the SEER*Stat software. We were, therefore, able to calculate statistics for the B-cell malignancies DLBCL and B-cell ALL, which require ICD-O-3 coding. 

Nearly all countries in our paper had national-level incidence statistics for NHL and MM, although prevalence and survival data for these malignancies were less complete. Data on DLBCL and FL were only available in the United States, the United Kingdom, Germany, and Australia, but supplementary data were available on diffuse NHL from several countries (Brazil, England, Ireland, and Germany). 

Since the course of leukemia varies by its tissue of origin and whether it is acute or chronic [17], the reporting of data by specific leukemia subtypes is preferable. Lymphoid leukemia incidence was often used as a proxy for ALL and CLL, however, and prevalence and survival data were scarce for all categories of lymphoid leukemia. In many developed countries (e.g., Canada, the United Kingdom, and Germany), only the prevalence of leukemia as a large diagnostic group was reported. France provided no national-level estimates of ALL incidence, except rates estimated using a statistical method based on unreported mortality data (Belot A and Mitchell M, personal communication with Université Lyon, January 27, 2011). In Germany, descriptive statistics were only available for leukemia as a large diagnostic group. Regional statistics for lymphoid leukemia were found in the Bavaria and Bremen population-based cancer registries (i.e., registries that collected data on every person with incident cancer within a defined geographic region). Italy, on the other hand, provided pooled data from its 28 regional cancer registries on adult and pediatric ALL and adult CLL. Among the Asian countries, only Japan had national-level data on ALL, whereas China and South Korea had national-level data on lymphoid leukemia.

The United States and the Republic of Ireland were the only countries with incidence data specific to adult B-cell ALL (1.0 [18] and 0.22 (S. Deady and M. Wagner, personal communication, with National Cancer Registry of Ireland, September 24, 2010), respectively, per 100,000 persons). In the United States, these data suggest that B-cell ALL comprises approximately 60% of all adult ALL [18]. The United States was the only country with statistics on pediatric B-cell ALL, with an incidence of 1.77 (95% CI, 1.70−1.84) per one million children (representing only 5% of pediatric ALLs) [18]. Of note, France, Germany, and Spain reported the incidence of mature B-cell lymphoid leukemias (International Classification of Childhood Cancer [ICCC] code Ia2) among children 0 to 14 years of age (1.5, 1.0, and 0.6 per million children per year, resp.); these data demonstrate that the vast majority of lymphoid leukemias in children are of the precursor cell type [19–21]. 

Cancer registries in some countries indicated that the provision of descriptive data on specific NHL and leukemia subtypes was not possible because of small population sizes and the associated uncertainty with small numbers. The Northern Ireland Cancer Registry, for example, could not provide statistics by NHL or leukemia subtypes because of small numbers (D. Donnelly and M. Wagner, personal communication with Northern Ireland Cancer Registry, September 27, 2010). The National Cancer Registry of the Republic of Ireland, on the other hand, provided these data after e-mail contact was initiated with the registry (S. Deady and M. Wagner, personal communication with National Cancer Registry of Ireland, September 24, 2010).

Other cancer registries could not provide the level of detail required for NHL and leukemia subtypes because the data were not coded appropriately to permit such analyses. Belot et al. [22], for example, noted that it is not currently possible to calculate statistics for various hematologic subtypes in France because of issues with reliably classifying cases using a consistent coding scheme. Similarly, the Office of National Statistics (which releases descriptive data for England) stated that they were only able to provide data by ICD-10 codes because of variability in the coding information provided by regional registries and issues with mapping ICD-O-3 (introduced in 2008) to ICD-O-2 (N. Jakomis and M. Wagner, personal communication with National Cancer Intelligence Center on Descriptive Cancer Statistics, October 11, 2010). ICD-10 codes do not allow for the calculation of statistics on B-cell ALL or DLBCL. We were able to capture statistics for FL with ICD-10 code C82 and for diffuse lymphoma with ICD-10 code C83, however. 

The ICD-O-3 coding system provides the most relevant information for descriptive epidemiologic statistics because malignancies are coded by cell lineage and maturity, as well as morphologic, genotypic, genetic, immunohistochemical, and clinical behavior. ICD-O-3 has been adopted by the US and most European countries, but the report of descriptive statistics by ICD-O-3 coding is limited by the recent conversion from ICD-O-2 in 2000 and the small numbers associated with these rare hematologic malignancies [1]. One author noted that, even considering the whole of Europe, some entities were too rare to calculate statistics by ICD-O-3 coding [23]. To address this issue, HAEMACARE, a project funded by the European Commission to improve the standardization of population-based data on hematologic malignancies, collected incidence data 2000–2002 from 48 European registries in 20 countries using a grouping system based on WHO recommendations and the ICD-O-3 morphology codes. Thus, categories represent cell lineage and prognosis, which are useful for epidemiologic and public health purposes [1]. The HAEMACARE coding system contains detailed information on mature B-cell malignancies, including DLBCL, FL, and CLL. In their first publication of incidence data from the HAEMACARE network, Sant et al. [1] reported European age-standardized incidence rates (per 100,000 persons) for DLBCL, FL, and CLL of 3.81 (95% CI, 3.73−3.89), 2.18 (95% CI, 2.12−2.24), and 4.92 (95% CI, 4.83−5.01), respectively [1]. Compared to these HAEMACARE European estimates, the incidence of DLBCL is considerably higher in the United States (6.83; See Table S3) and the incidence of FL is higher in both the United States (3.7) and Australia (4.1; See Table S3). However, the HAEMACARE European estimate of CLL incidence is similar to rates observed in the United States (5.0), Canada (5.0), and Australia (4.9; See Table S7). Sant et al. [1] noted geographic variability in the incidence of hematologic malignancies across Europe, which they attributed to differences in diagnostic and registration criteria. They concluded that the quality of the data still requires improvement. This first publication from the HAEMACARE network suggests that data on DLBCL, FL, and CLL are increasingly available in certain registries across Europe, but that cancer registries are reluctant to regularly publish and/or provide these statistics.

Another limitation of the diagnostic classification system used for reporting cancer statistics is the use of the ICCC for childhood cancers. In the third version of the ICCC, ALL is categorized along with other lymphatic leukemias in the category ICCC-1a, which includes morphology codes 9820, 9823, 9826, 9827, 9831–9837, 9940, and 9948 [24]. Although the vast majority of lymphatic leukemias in childhood are acute in nature, estimates using ICCC-1a only provide a proxy for actual ALL rates [17].

### 3.2. Variability in the Methods Used by Cancer Registries

There was wide variation between countries in the methods used for estimating national-level descriptive statistics. Some countries used direct estimates from national cancer registries, collecting information on all cancer diagnoses nationwide into 1 registry (i.e., Wales, Northern Ireland, Republic of Ireland, Scotland, and South Korea) or a coordinated group of regional registries (i.e., England, Canada, and Australia). Other countries calculated estimates of the national-level experience using statistical methods to extrapolate data from regional registries to the national level (i.e., United States, France, Japan, and China). Another group of European countries estimated the national-level cancer experience by pooling data from regional registries (i.e., Italy, Germany, and Spain). This variability in the methods used to estimate national-level statistics limits the comparability of statistics between countries. 

One statistical method used to predict cancer incidence at the national-level is the use of mortality rates (which are often readily available nationwide) as a geographic correlate of incidence. Observed incidence and mortality data are modeled in this approach using age-cohort techniques to provide estimated incidence rates. This method was recently used to estimate cancer incidence in France and China [22, 25] and is frequently used by GLOBOCAN [26]. While this method is known to be generally reliable, there are some questions about the representativeness of the data used [25]. However, the reliability of this method to estimate the incidence cancers with high survival rates (e.g., pediatric ALL) is less clear. 

The vast majority of registries were population-based, but some countries had registries that collected data only on cancer patients seen at a particular hospital (i.e., hospital-based registries). Hospital-based registries are limited compared with population-based cancer registries because the source population is not well-defined. Brazil has several hospital-based cancer registries [27], and South Korea has a nationwide, hospital-based cancer registry (the Korea Central Cancer Registry) that reportedly captures 90% of all cancer cases nationwide [28]. 

Some countries have a separate registry for childhood cancers. The National Registry of Childhood Tumors is a population-based cancer registries of malignancies and benign brain tumors diagnosed in children living in England, Wales, or Scotland [29]. The French National Registry of Childhood Hematopoietic Malignancies has recorded all cases of hematologic malignancies, including lymphoma, since 1990; the French National Registry of Childhood Solid Tumors has recorded all cases of solid tumors, except lymphomas, since 2000 [19]. The German Childhood Cancer Registry has collected data on cancer cases nationwide, with an estimated coverage of about 95% [30]. Finally, the Australian Pediatric Cancer Registry has provided full coverage of all Australian states and territories since 1983 [31]. 

### 3.3. Implications of Regional Cancer Registration

A substantial number of countries do not have a nationwide cancer registration program. Rather, cancer registration consists of a group of regional cancer registries, collecting complete data on cancer cases in their respective geographic regions and coordinated by an overarching network. The level of standardization and coordination between these regional registries varies substantially by country. In the United States, SEER currently collects and publishes cancer data in a standardized fashion from 17 population-based cancer registries covering approximately 28% of the US population [18]. There are 21 regional cancer registries in France coordinated under the Association of French Cancer Registries (FRANCIM) that cover approximately 16% of the population [22]. In Italy, there are 28 registries that cover approximately 26% of the population coordinated through the Italian Association of Cancer Registries (AIRTUM) [32]. In Germany, there are regional registries in 13 states and 1 administrative district, coordinated through the Robert Koch Institute [30]. Japan has approximately 30 population-based cancer registries that are coordinated through the Japan Cancer Surveillance Research Group [33]. Some developed countries still lack coordination of their regional registries, which limits what can be done to predict the national-level experience of cancer. Spain, for example, currently has 13 regional registries that cover approximately 27% of the population, but no network that coordinates and standardizes these registries; a network is currently under development, however [34]. 

In less developed countries, where financial, resource, and logistical challenges dominate, coverage is typically limited to metropolitan areas, and differences between regional registries preclude the development of national estimates. Substantial efforts are being made, however, to organize and standardize these regional registries into a coordinated network. In Brazil, for example, efforts are being made to collect data from an expanding network of regional registries and to improve data quality to support nationwide cancer prevention and planning. Epidemiology surveillance was formally organized and standardized in 1999 and now includes 22 population-based cancer registries, most of which include capital cities and surrounding areas. These population-based cancer registries vary in the source (hospital, laboratory, etc.) and quality of their data [27]. Because of this variation, pooled data cannot be reported and statistics are provided only by individual population-based cancer registries for Brazil. The age-adjusted incidence of NHL, for example, varied from 2.0 to14.1 per 100,000 among males in the ten population-based cancer registries with more than 2 years of data [27]. The authors of the latest report cautioned that these results are not generalizable to Brazil as a whole because the population-based cancer registries are still of “heterogeneous quality, validity, and completeness” [27]. Underreporting also remains a serious concern in Brazil and other less developed countries. Despite these limitations, some Brazilian population-based cancer registries provided data on detailed diagnostic categories—diffuse NHL, follicular lymphoma, and pediatric ALL. However, although incidence data were readily available from Brazilian population-based cancer registries, more complex data (prevalence and survival) were not. Similarly, India has 26 population-based cancer registries and 6 hospital-based cancer registries coordinated under the National Cancer Registry Programme network and covering 7% of the population [35]; statistics are only reported by population-based cancer registries. China also has no national-level coordination of its 50 population-based cancer registries, which cover 5.7% of the country [36].

### 3.4. Cancer Registry Evolution

This survey represents the status of data that were available as of mid-July 2011 using comprehensive methods. It is clear from our paper, however, that the breadth and specificity of data reported by cancer registries is continually evolving, and this evolution will ultimately entail the reporting of data at the national level for detailed cancer subtypes. In France, for example, national level data were reported for the first time in 2003 for cancer incidence [37], in 2006 for cancer survival [38], and in 2008 for cancer prevalence [39]. Lacour et al. [19] described the incidence of childhood cancers nationwide in France for the first time in 2010. Population-based cancer registration in Germany improved considerably after the Federal Cancer Registry Data Act of 2009, which called on all federal states to report complete data to the German Centre for Cancer Registry Data at the Robert Koch Institute, thus expanding the breadth of cancer statistics in Germany from data reported only by the Saarland Cancer Registry to pooled analyses of data from cancer registries meeting the minimum requirements for data completeness. Current estimates of cancer incidence are based on data from 13 states and 1 administrative district [30], and the GEKID Cancer Survival Working Group recently published their first comprehensive monitoring of cancer survival in Germany based on a collaboration between 13 population-based cancer registries [40].

### 3.5. General Data Patterns

Some basic patterns in the descriptive epidemiology of B-cell malignancies emerged from reviewing the available data. Specifically, the incidence rates of the B-cell neoplasms included in this paper were generally higher in the United States, Australia, and the European Union-5, compared with Asia and Brazil (possibly reflecting differences in the ascertainment of cases). Further, the incidence of NHL was generally higher in men, and NHL survival rates were slightly higher in women (See Table S2). The higher incidence of hematologic malignancies in men versus women may be explained by the traditionally higher exposures of men to occupational and environmental carcinogens [1].

### 3.6. Limitations

Many cancer registries reported that the requested statistics were not readily available or were available at a cost (e.g., additional data were available at a cost from the Office of National Statistics, the Welsh Cancer Intelligence Service, and the Australian Institute of Health and Welfare). The acquisition of data at a cost was beyond the scope of this paper. It is, therefore, possible that statistics beyond those reported in Table 3 are available. 

Some attempts to communicate with key experts and cancer registries failed, thus limiting our understanding of the true availability of certain data (Table 2). For example, our attempts to contact the Northern and Yorkshire Cancer Registry and Information Service (NYCRIS) and the Cote d'Or Hematological Malignancies Registry (the lead registries for hematologic malignancies in England and France, resp.) yielded no responses. We were also unable to establish contact with cancer registries in Italy, Japan, and South Korea.

We limited the data we gathered to diagnoses post-2000 to allow for representation of the availability of recent descriptive statistics. Some data were only available, however, for diagnoses that occurred in the late 1990s (e.g., survival data in Ireland and France and pediatric ALL incidence in Scotland). This paper was also limited to select countries, which precludes a full evaluation of the worldwide availability of data and an analysis of geographic differences. A comparison of statistics across countries is also limited by differences in the structure of cancer registries, age standardization methods, methods for estimating the statistics (e.g., period estimates for survival), and the year(s) of diagnosis. Furthermore, the comparison of hematologic malignancies across time and place was significantly limited by regional differences in disease classification systems. 

This paper was also limited by reports of cancer data in non-English languages. Translations of non-English language material were restricted to languages in which the research team had proficiency and were not carried out in a systematic manner. Thus, it is possible that additional data is readily accessible in other languages. 

Another limitation of this paper is the constantly evolving nature of descriptive cancer statistics. Updates are published continuously by cancer registries, and investigators are frequently publishing manuscripts with additional, relevant epidemiologic data. We attempted to capture the most recent data available as of approximately mid-July 2011. At the time of the publication of this study, however, we were aware of numerous updates, namely the release of updated SEER data [16]. In addition, the NYCRIS was planning to publish a report on hematologic malignancies for all English cancer networks in 2011 [41].

## 4. Conclusions

This paper used a novel approach to gathering descriptive epidemiologic statistics on cancer by combining information from the biomedical literature, Internet-based query systems and reports, and direct contact with cancer registry representatives and key experts to provide the most up-to-date, comprehensive picture of a particular malignancy type.

This paper highlights the importance of international coordination between cancer registries (e.g., HAEMACARE) to promote standardization in data collection and reporting. This is particularly important for rare malignancies that require complicated diagnostic evaluations and reporting. International coorindination and standardization of cancer registration is recommended in South America and Asia. We also recommend continued coordination and standardization between regional registries in countries such as Brazil, Spain, and India to promote the development of nationwide estimates. 

This novel approach provided a more comprehensive database of the current patterns of B-cell malignancies compared with a simple literature search. In addition, gaps were identified that highlight the need for more detailed reporting to cancer registries and the importance of developing assumptions to estimate the burden of specific B-cell malignancies in the interim. Because research indicates that cellular lineage affects prognosis, treatment, and etiology, cancer registries should continue to work to improve reporting of detailed diagnostic information on malignancies.

## Supplementary Material

The supplementary material includes the following Tables: Supplementary Table 1 details the PubMed search strategy; and Supplementary Tables 2 to 7 provide the most recent statistics by country and statistic type for NHL, DLBCL and FL, adult ALL, pediatric ALL, MM, and CLL, respectively.

## Figures and Tables

**Table 1:** Classification of B-cell neoplasms* [5].

Precursor B-cell neoplasm	
**Precursor B-lymphoblastic leukemia/lymphoma**	
Mature (peripheral) B-cell neoplasms	
**Diffuse large B-cell lymphoma (DLBCL)**	
**Follicular lymphoma (FL), follicle center**	
**Plasma cell (or multiple) myeloma (MM)/plasmacytoma**	
**B-cell chronic lymphocytic leukemia/small lymphocytic** **lymphoma**	
B-cell prolymphocytic leukemia	
Lymphoplasmacytic lymphoma	
Splenic marginal zone B-cell lymphoma	
Nodal marginal zone lymphoma	
Extranodal marginal zone B-cell lymphoma of mucosa-associated lymphoid tissue (MALT) type	
Hairy cell leukemia	
Mantle cell lymphoma	
Burkitt lymphoma/Burkitt cell leukemia	

*Bold neoplasms were included in our paper.

**Table 2:** Cancer registries and organizations contacted to obtain descriptive epidemiologic statistics on B-Cell malignancies by country.

Country	Agency or registry	Contact established?*	Additional statistics provided?
Australia	Australian Paediatric Cancer Registry	Yes	Yes
Australia	Australian Institute of Health and Welfare	Yes	No^†^
Brazil	Instituto Nacional de Cancer	Yes	No^‡^
Canada	Statistics Canada	Yes	No^‡§^
Canada	Canadian Childhood Cancer Surveillance and Control Program	Yes	No^‡^
China	Cancer Institute and Hospital of the Chinese Academy of Medical Sciences/Chinese National Center for Cancer Registries	Yes	No^‡^
England	Office of National Statistics	Yes	No^†^
England	Northern and Yorkshire Cancer Registry and Information Service	No	—
France	National Registry of Childhood Haematopoietic Malignancies	No	—
France	Institut de Veille Sanitaire	Yes	No^‡^
Germany	German Centre for Cancer Registry Data at the Robert Koch Institute	Yes	No^‡^
India	National Cancer Registry Programme	Yes	Yes
Republic of Ireland	National Cancer Registry in Ireland	Yes	Yes
Northern Ireland	Northern Ireland Cancer Registry	Yes	Yes
Italy	Italian Association of Cancer Registries	No	—
Japan	National Cancer Center	No	—
Japan	Japan Association of Cancer Registries	No	—
South Korea	Korea Central Cancer Registry	No	—
South Korea	College of Medicine, Korea University	No	—
Spain	Spanish National Childhood Cancer Registry	Yes	Yes
Spain	National Center of Epidemiology, Instituto de Salud Carlos III	Yes	No^‡^
United Kingdom	National Registry of Childhood Tumors	Yes	No^‡^
United Kingdom	Cancer Research UK	Yes	No^‡^
Wales	Welsh Cancer Intelligence Service	Yes	No^†^

*All contacts were identified by reviewing contact information included in pertinent publications and information available online.

^†^Replied that additional statistics only available at a cost.

^‡^Directed to existing material.

^§^Replied that requested statistics not readily available.

**Table 3:** Summary of available epidemiologic data by outcome and country.

	Total NHL			DLBCL and FL			Adult ALL			Pediatric ALL			MM			CLL		
North and South America	I	P	S	I	P	S	I	P	S	I	P	S	I	P	S	I	P	S

United States	*✓*	*✓*	*✓*	*✓*	*✓*	*✓*	*✓*	*✓*	*✓*	*✓*	*✓*	*✓*	*✓*	*✓*	*✓*	*✓*	*✓*	*✓*
Canada	*✓*	*✓*	*✓*				*✓*		*✓*	✓*		✓*	*✓*	*✓*	*✓*	*✓*		*✓*
Brazil	*✓* ^†^			*✓* ^†^			*✓* ^∗†^			*✓* ^∗†^			*✓* ^†^			*✓* ^∗†^		

European Union-5	I	P	S	I	P	S	I	P	S	I	P	S	I	P	S	I	P	S

United Kingdom	*✓*	*✓*	*✓*	*✓*			*✓*			*✓*		*✓*	*✓*	*✓*	*✓*	*✓*		*✓*
England	*✓*	*✓*	*✓*	*✓* ^†^			*✓*						*✓*	*✓*	*✓*	*✓*		
Ireland	*✓*			*✓*									*✓*		*✓*			
Republic of Ireland	*✓*		*✓*	*✓*		*✓*	*✓*		*✓*	*✓*			*✓*		*✓*	*✓*		*✓*
Northern Ireland	*✓*	*✓*	*✓*	*✓*			*✓*		*✓*				*✓*	*✓*	*✓*	*✓*		*✓*
Scotland	*✓*	*✓*	*✓*				*✓*	*✓*					*✓*	*✓*	*✓*	*✓*	*✓*	
Wales	*✓*	*✓*	*✓*				*✓*	*✓*	*✓*	✓*			*✓*	*✓*	*✓*	*✓*	*✓*	*✓*
France	*✓*	*✓*	*✓* ^†^							✓*		*✓*	*✓*	*✓*	*✓*	*✓*		
Germany	*✓*	*✓*	*✓*	*✓* ^†^			*✓* ^†^			✓*		✓*	*✓*		*✓*	*✓* ^†^		
Italy	*✓*	*✓*	*✓*				*✓*	*✓*	✓*	*✓*		*✓*	*✓*	*✓*	*✓*	*✓*	*✓*	✓*
Spain	*✓*		*✓* ^†^				*✓* ^∗†^			✓*		*✓*	*✓*			*✓* ^∗†^		

Asia	I	P	S	I	P	S	I	P	S	I	P	S	I	P	S	I	P	S

China	*✓*		*✓* ^†^				✓*		*✓* ^∗†^	*✓* ^∗†^			*✓*		*✓* ^†^	✓*		*✓* ^∗†^
Japan	*✓*						*✓*						*✓*					
South Korea	*✓*	*✓*	*✓*				✓*	✓*	*✓* ^∗†^	✓*	✓*		*✓*	*✓*	*✓*	✓*	✓*	*✓* ^∗†^
India	*✓* ^†^		*✓* ^†^				*✓* ^∗†^			*✓* ^∗†^		*✓* ^∗†^	*✓* ^†^			*✓* ^∗†^		

Australia	I	P	S	I	P	S	I	P	S	I	P	S	I	P	S	I	P	S

Australia	*✓*		*✓*	*✓*			*✓*			✓*		*✓*	*✓*			*✓*		

ALL: acute lymphoblastic leukemia; CLL: chronic lymphocytic leukemia; DLBCL: diffuse large B-cell lymphoma; FL: follicular lymphoma; I: incidence; MM: multiple myeloma; NHL: non-Hodgkin's lymphoma; P: prevalence; S: survival.

*Data on lymphoid leukemia were used as a proxy for ALL and CLL.

^†^Regional estimates were used a proxy for national estimates.
