# Supplementary material for: A Multisource Approach to Improving Epidemiologic Estimates: Application to Global B-Cell Malignancies
Source: ISRN Oncol. 2012 Dec 31;2012:129713. doi: 10.5402/2012/129713 (PMC3549359; doi:10.5402/2012/129713)
Supplement: Supplementary file 1 — The supplementary material includes the following Tables: Supplementary Table 1 details the PubMed search strategy; and Supplementary Tables 2 to 7 provide the most recent statistics by country and statistic type for NHL, DLBCL and FL, adult ALL, pediatric ALL, MM, and CLL, respectively. [file 129713.f1.docx]

**Supplementary Table 1. PubMed Search Summary**

| **Search Number** | **Search Descriptor** | **Search String** | **Search Limits** | **Publication Date Limit** |
| --- | --- | --- | --- | --- |
| 1 | Geographic region | United States OR Canada OR North America OR Australia OR Japan OR India OR China OR Korea OR Spain OR Italy OR Germany OR France OR England OR UK OR Ireland OR Scotland OR Wales OR Europe | Humans | January 1, 2000– July 13, 2011 |
| 2 | Terminology | (incidence OR survival OR prevalence OR "cancer patterns" OR "cancer trends" OR "descriptive statistics" OR epidemiology OR burden) AND ("B-cell malignancies" OR lymphoma OR myeloma OR cancer OR leukemia OR lymphohematopoietic OR malignancies) NOT (breast OR colorectal OR prostate OR lung OR colon) | Humans; title | January 1, 2000– July 13, 2011 |
| 3 | Geographic region AND terminology | <<Search 1>> AND  <<Search 2>> | Humans | January 1, 2000–July 13, 2011 |

**Supplementary Table 2**. **Incidence, Prevalence, and Survival Associated With NHL* by Country^†^**

|  | **Incidence per 100,000 (95% CI)**  **[Year(s) of Diagnosis]** | **Prevalence per 100,000 (95% CI) [Date of Estimate]** | **Relative Survival, % (95% CI)**  **[Year(s) of Diagnosis]** |
| --- | --- | --- | --- |
| **North and South America** | |  |  |
| United States | All=19.79 (19.47–20.11)a; M=24.03 (23.51–24.57)a; F=16.42 (16.03–16.82)a  [2007] [18] | **1 y:** All=13.7; M=15.9; F=11.8  **5 y:** All=55.9; M=64.1; F=49.0  **7 y:** All=71.9; M=82.1; F=63.1  [July 1, 2007] [18] | **1 y:** All=79.9 (79.1–80.6); M=79.4 (78.3–80.4); F=80.4 (79.3–81.5)  [2006] [42]  **3 y:** All=71.5 (71.1–71.9); M=70.2 (69.7–70.7); F=72.9 (72.4–73.5)  [2001–2006] ^[42]^  **5 y:** All=63.5 (62.8–64.3); M=61.9 (60.9–62.9); F=65.4 (64.3–66.5)  [2000–2001] [18] |
| Canada | All=16.9 (16.4–17.3)a; M=20.4 (19.7–21.1)a;  F=13.8 (13.3–14.3)a  [2007] [43] | **2 y:** All=18.9b; M=21.7b; F=16.3b  **5 y:** All=39.0b; M=44.2b; F=34.1b  **10 y:** All=60.4b; M=67.5b; F=53.9b  [Jan. 1, 2005] [44] | **1 y:** 78 (77–79)  **3 y:** 68 (68–69)  **5 y:** 63 (62–64)  **10 y:** 54 (53–55)  [2004–2006] [45] ^‡,§^ |
| Brazil | Goiânia [1996–2000] [46]: M=7.7b; F=5.7b  Salvador [1997–2001] [46]: M=4.6b; F=3.8b  João Pessoa [1999–2000] [46]: M=4.3b; F=3.2b | Not reported | Not reported |
| **European Union–5** | |  |  |
| United Kingdom | All=15.1c; M=17.7c; F=12.8c  [2008] [47] | **1 y:** All=10.4 (10.1–10.6)c; M=12.1 (11.7–12.5)c; F=8.8 (8.5–9.1)c  **5 y:** All=39.8 (39.3–40.2)c; M=45.6 (44.8–46.3)c; F=34.6 (34.0–35.2)c  **10–y:** All=62.6 (62.0–63.2)c;  M=71.1 (70.2–72.1)c; F=54.9 (54.2–55.7)c  [Dec. 31, 2006] [48] | **5 y:** All=51.2; M=49.6; F=53.3  **10 y:** All=41.3; M=40.7; F=42.4  [2000–2002] [13]^‡^ |
| England | All=15.1c; M=17.7c; F=12.9c  [2008][47] | **1 y:** All=10.4 (10.1–10.6)c; M=12.1 (11.7–12.6)c; F=8.8 (8.5–9.1)c  **5 y:** All=39.7 (39.2–40.2)c; M=45.6 (44.8–46.4)c; F=34.4 (33.7–35.1)c  **10 y:** All=62.6 (62.0–63.3)c; M=71.5 (70.5–72.5)c; F=54.7 (53.9–55.6)c  **20–y:** All=83.5 (82.7–84.2)c;  M=96.1 (94.9–97.2)c; F=72.2 (71.2–73.1)c  [Dec. 31, 2006] [48] | **1 y:** All=72.8 (72.3–73.3); M=72.9 (72.2–73.6);  F=72.8 (72.0–73.5)  [2000–2004] [49] **5 y:** M=59.3 (58.4–60.3)e; F=64.2 (63.3–65.1)e  [2004–2008] [50]  **10 y:** All=41.3; M=39.1; F=43.5  [2000–2002] [13]^§^ |
| Ireland | All=13.9c; M=15.6c; F=12.5c  [2000–2004] [51] | Not reported | Not reported |
| Northern Ireland | All=12.1 (10.7–13.5)b; M=13.5 (11.4–15.6)b; F=10.9 (9.1–12.7)b  [2009] [52]  All=16.8 (14.9–18.6)c; M=18.8 (16.0–21.6)c; F=15.1 (12.7–17.5)c  [2009] [52] | **1 y:** All=10.1 (8.7–11.6)c; M=12.1 (9.8–14.4)c; F=8.5 (6.6–10.4)c  **5 y:** All=41.6 (38.7–44.6)c; M=46.2 (41.7–50.8)c; F=37.6 (33.7–41.5)c  **10 y:** All=67.7 (63.9–71.4)c; M=72.9 (67.2–78.7)c; F=63.1 (58.1–68.1)c  [Dec. 31, 2006] [48] | **1 y:** All=72.3 (68.8–75.8); M=72.5 (67.5–77.5); F=72.1 (67.2–76.9)  [2001–2003] [52]  **3 y:** All=56.0 (52.6–59.5); M=55.5 (50.5–60.5); F=56.5 (51.7–61.3)  [1997–2000] [52]  **5 y:** M=48.6 (43.3–53.9); F=52.9 (47.8–58.0) [1997–2000] [52] |
| Republic of Ireland | All=13.9c; M=15.7c; F=12.2c [2007–2009][53] | Not reported | **5–y**: All=61.2e  [2005–2008] [54] |
| Scotland | All=10.1 (9.4–10.8)b; M=11.6 (10.5–12.7)b; F=9.0 (8.1–9.9)b  [2008] [55]  All=14.2 (13.2–15.1)c; M=16.3 (14.8–17.8)c; F=12.0 (10.8–13.2)c  [2008] [55] | **Prevalence rate (proportion [%])**  **<1 y:** All=14.5 (0.015)d; M=15.0 (0.015)d; F=14.1 (0.014)d  **1–5 y:** All=40.8 (0.041)d; M=42.6 (0.043)d; F=39.2 (0.039)d  **5–10 y:** All=30.6 (0.031)d; M=30.3 (0.030)d; F=30.9 (0.031)d  **10–20 y:** All=28.2 (0.028)d; M=29.8 (0.030)d; F=26.7 (0.027)d  [Dec. 31, 2007] [55] | **1 y:** All=73.6; M=72.0; F=75.2 **3 y:** All=64.1; M=62.3; F=65.8  **5 y:** All=59.7; M=58.3; F=61.0  [2003–2007] [55] |
| Wales | M=16.2 (14.4–18.2)c; F=11.6 (10.2–13.2)c  [2009][56]  All=15.8c  [2008][47]  M=11.6 (10.2–13.2)b; F=8.3 (7.2–9.7)b [2009] [56] | **Prevalence rate (proportion [%])**  **<1 y:** All=16.6 (0.02)d; M=18.1 (0.02)d; F=15.3 (0.02)d  **1–5 y:** All=44.1 (0.04)d; M=48.3 (0.05)d; F=40.1 (0.04)d  **5–10 y:** All=29.8 (0.03)d; M=31.8 (0.03)d; F=27.9 (0.03)d  **10–20 y:** All=25.1 (0.03)d; M=25.6 (0.03)d; F=24.7 (0.02)d  [Dec. 31, 2008] [57] | **1 y:** All=76.1; M=75.6; F=76.8  **5 y:** All=62.3; M=60.0; F=64.9  [2004–2008] [58]^‡^ |
| France | M=12.1b; F=8.2b  [2005][22]  M=15.9c,f; F=10.9c,f  [2008] [26] | **5 y**: M=38.1c; F=29.0c  [Dec. 31, 2002] [39] | **5 y**: All=57.4e,g; M=57.4e,g; F=58.6e,g  [2000–2002] [13]^‡^ |
| Germany | M=9.2b; F=7.2b  [2006][59]  M=13.0c,f; F=9.9c,f  [2008] [26] | **Proportion (%) 1 y:** M=0.01; F=0.01  **2 y:** M=0.03; F=0.02  **3 y:** M=0.04; F=0.03  **5 y:** M=0.05; F=0.05 **10 y:** M=0.08; F=0.08 [2004] [60] | **5–y**: 62.8e  [2002–2006][40]  **5 y:** All=61.9e,g; M=60.4e,g; F=65.9 e,g  [2000–2002] [13]^‡^ |
| Italy | M=16.4c,f; F=12.3c.f [2008] [26] | **<2 y:** All**=**33; M=37; F=30 **<5 y:** All**=**69; M=76; F=64 **<10 y:** All**=**113; M=121; F=106 **<15 y**: All=139; M=147; F=132 **<20 y**: All=154; M=161; F=148  [Jan. 1, 2006] [61] | **1 y:** All=76; M=76; F=76 **3 y:** All=64; M=64; F=65  **5 y:** All=59 (58–60); M=59 (57–60); F=60 (59–61)  [not reported] [62] |
| Spain | All=12.0c; M=14.2c; F=10.1c  [2000–2004][63] | Not reported | **5 y:** All=51.9g; M=40.9g; F=63.2g  [2000–2002] [13]^‡^ |
| **Asia** | |  |  |
| China | All=4.8d  [2006]^\|\|^  M=3.0b,f; F=1.5b,f  [2005] [25] | Not reported | **Hong Kong:**  **1–y:** All=69.5 **3–y:** All=58.8  **5– y:** All=56.1; M=54.9; F=57.7  [1996–2001] [64] **Qidong:  1–y:** All=27.0 **3–y:** All=18.0  **5 y:** All=13.9; M=14.9; F=12.5  [1992–2000] [36] |
| Japan | M=6.51b; F=3.78b  [not reported][65] | Not reported | Not reported |
| South Korea | All=5.8a; M=7.0a; F=4.9a All=5.5b; M=6.7b; F=4.6b  [2008] [66] | **5 y:** All=18.3a; M=21.1a; F=15.8a **5 y:** All=17.3b; M=20.2b; F=14.8b [2008] [66] | **5 y:** All=62.8; M=61.0; F=65.0  [2004–2008] [66] |
| India | Imphai West District: M=6.8b; F=3.0b  Delhi: M=5.8b; F=3.0b  Bangalore: M=5.6b; F=3.2b  Chennai: M=4.8b; F=2.7b  Mumbai: M=4.8b; F=2.8b  Thiruvananthapuram: M=4.6b; F=2.7b  Bhopal: M=0.0b; F=2.2b  Kollam: M=3.9b; F=1.7b  Manipur State: M=3.8b; F=2.2b  Pune: M=3.5b; F=2.0b  Nagpur: M=3.2b; F=1.6b  Kamrup Urban District: M=3.0b; F=1.7b  Manipur State, excl. Imphal West: M=3.0b; F=1.9b  Ambedabad Urban: M=2.9b; F=2.0b  Kolkata: M=2.5b; F=2.1b  Aizawi District: M=2.4b; F=2.2b  Aurangabad: M=2.2b; F=1.5b  Ahmedabad Rural: M=1.8b; F=0.8b  Barshi Expanded: M=1.8b; F=0.8b  Mizoram State: M=1.7b; F=1.2b  Cachar District: M=1.7b; F=0.3b  Barshi Rural: M=1.7b; F=1.0b  Dibrugarh District: M=1.6b; F=1.0b  Mizoram State, Excl. Aizawi: M=1.3b; F=0.5b  R Sikkim State: M=1.1b; F=0.4b  [2006–2008] [67] | Not reported | **Barshi:  1 y:** All=42.5 **3 y:** All=30.0  **5 y:** All=25.4; M=20.8; F=33.0  [1993–2000] [68] |
| **Australia** |  |  |  |
| Australia | All=18.0a; M=21.1a; F=15.4a  All=13.5b; M=15.6b; F=11.4b  [2007] [69] | Not reported | **1 y:** All=77.9 (77.3–78.4); M=78.1 (77.4–78.9); F=77.5  **5 y:** All=62.1 (61.3–62.8); M=61.6 (60.6–62.6); F=62.6 (61.5–63.6)  **10 y:** All 51.6; M=51.6; F=51.6  [1996–2004] [70,71] |

F=female; M=male; NHL=non-Hodgkin lymphoma.

*The following diagnostic codes defined NHL in each country: Australia (ICD-10 C82-85); Brazil (ICD-10 C82-85 and C96); China (ICD-10-C82-85 and C96); France (rates standardized to world population: ICD-0-3 95903-95963, 96703-97193, 97273-97293, 98323-98343; rates standardized to European population: ICD-10 C82-86 and C96); Germany (CID-10-C82-85); India (ICD-10 C82-C85 and C96); Italy (ICD-10 C82-C85 and C96); South Korea (not reported); Spain (ICD-10-C82-C85 and C96); United Kingdom (ICD-10 C82-C85 and C96); England (ICD-10 C82-C85 and C96); Northern Ireland (ICD-10-C82-C85 and C96); Scotland (ICD-10-C82-C85).

^†^Countries not listed in this table did not provide results for NHL. Regional estimates are provided when national estimates were unavailable.

^‡^Survival rates were predicted by period survival analysis.

^§^Quebec is excluded from the survival analysis.

^||^L. Watson, M. Mitchell, personal communication, October 19, 2010.

^a^Incidence rates are age-adjusted to the national population.

^b^Incidence rates are age-adjusted to the world standard population.

^c^Incidence rates are age-adjusted to the European population.

^d^It is unclear which population was used for age adjustment.

^e^Survival results reported for persons 15-99 years of age.

^f^Incidence rates are estimated.

^g^Survival results based on select regional data.

**Supplementary Table 3. Incidence, Prevalence, and Survival of DLBCL and FL by Country^*^**

|  | **Incidence per 100,000 of DLBCL**  **(95% CI)^†‡^**  **[Year(s) of Diagnosis]** | **Incidence per 100,000 of FL**  **(95% CI)^§^**  **[Year(s) of Diagnosis]** |
| --- | --- | --- |
| **North and South America** | |  |
| United States | All=6.83 (6.65–7.02)a; M=8.46 (8.15–8.78)a; F=5.48 (5.26–5.71)a  [2007] [18] | All=3.7 (3.56–3.84)a; M=3.99 (3.79–4.21)a; F=3.48 (3.3–3.66)a  [2007] [42] |
| Brazil | *Belo Horizonte (2000) [46]: M=4.8b; F=3.01b*  *Goiânia (1996–2000) [46]: M=2.70b; F=1.53b*  *João Pessoa (1999–2000) [46]: M=1.91b; F=2.42b*  *Salvador (1997–2001) [46]: M=0.89b; F=0.78b* | Belo Horizonte [2000] [46]: M=0.14b; F=0.56b  Goiânia [1996–2000] [46]: M=0.60b; F=0.69b  Salvador [1997–2001] [46]: M=0.17b; F=0.01b |
| **European Union – 5** | |  |
| United Kingdom | All=3.67c  [2000–2002] [1] | All=2.19c  [2000–2002] [1] |
| England | *Northern and Yorkshire Cancer Registries: All=7.1c; M=9.0c; F=5.2c*  *[2009] [72]* | Northern and Yorkshire Cancer Registries: All=2.3c; M=2.2c; F=2.4c [2009] [72] |
| Ireland | *M=4.6 (4.2–5.0)d; F=3.1 (2.8–3.4)d*  *[2000–2004] [51]* | M=2.1 (1.8–2.3)d; F=2.1 (1.9–2.4)d  [2000–2004] [51] |
| Republic of Ireland | *All=5.3d; M=6.3d; F=4.3d*  *[2007–2009] [73]* | All=3.1d; M=3.2d; F=3.0d  [2007–2009] [73] |
| Northern Ireland | *M=3.4 (2.8–3.9)d; F=2.3(1.9–2.8)d*  *[2000–2004] [51]* | M=2.3 (1.9–2.8)d; F=2.3 (1.8–2.7)d  [2000–2004] [51] |
| Germany | *Munster PBCR: M=1.3b, 1.8c; F=2.2b, 2.8c*  *[2008] [74] Westfalen–Lippe region: M=3.6b, 4.9c; F=2.5b, 3.5c*  *[2008][74]*  *Hamburg PBCR: M=6.1b, 8.0c; F=2.7b, 3.5c*  *[2009] [75]*  *Lower Saxony PBCR: M=7.5b, 5.4c; F=4.4b, 3.2c*  *[2008] [76]*  *Schleswig–Holstein PBCR: M=7.8b, 5.9c; F=5.6b, 3.9c*  *[2008] [76]* | Munster PBCR: M=3.1b, 4.1c; F=1.9b, 2.8c  [2008] [74]  Westfalen–Lippe region: M=1.4b, 1.9c; F=1.8b, 2.5c  [2008] [74]  Hamburg PBCR: M=2.3b, 3.2c; F=1.6b, 2.2c  [2009] [75]  Lower Saxony PBCR: M=2.6b, 1.9c; F=2.6b, 1.9c  [2008] [76]  Schleswig–Holstein PBCR: M=2.8b, 2.0c; F=2.4b, 1.7c  [2008] [76,77] |
| **Australia** |  |  |
| Australia | All=6.2 (5.8–6.5)a; M=7.5 (7.0–8.1)a; F=5.0 (4.6–5.4)a [2007] [78] | All=4.1 (3.8–4.4)a; M=4.3 (3.9–4.7)a; F=3.9 (3.5–4.3)a [2007] [78] |

|  | **Prevalence of DLBCL per 100,000**  **(95% CI)**  **[Date of Estimate]** | **Prevalence of FL per 100,000**  **(95% CI)**  **[Date of Estimate]** |
| --- | --- | --- |
| **North and South America** | |  |
| United States | **1 y:** All=4.5; M=5.2; F=3.8  **7 y:** All=25; M=26; F=19.5  [July 1, 2007] [18] | **1 y:** All =3.0; M=3.2; F=2.9  **7 y:** All=16.8; M=17.8; F=16.0  [July 1, 2007] [18] |
|  | **Relative Survival of DLBCL, % (95% CI)**  **[Year(s) of Diagnosis]** | **Relative Survival of FL, % (95% CI)**  **[Year(s) of Diagnosis]** |
| **North and South America** | |  |
| United States | **1 y:** All=70.5 (69.3-71.6); M=70.2 (68.6-71.8); F=70.8 (69.0-72.4)  [2006] [18]  **5 y:** All=58.1 (57.1-59.2); M=57.2 (55.8-58.6); F=59.2 (57.6-60.7)  [2000-2001] [18] | **1 y:** All=93.3 (92.3-94.2); M=93.5 (92.0-94.8); F=93.0 (91.5-94.3)  [2006] [18]  **5 y:**All=82.8 (81.5-84.0); M=81.5 (79.6-83.2); F=84.1 (82.2-85.7)  [2000-2001] [18] |
| **European Union–5** |  |  |
| United Kingdom | **5-yr:** 49.2 (47.0-51.4) [2000-2002] [23] | **5-yr:** 73.1 (70.5-75.8) [2000-2002] [23] |
| Republic of Ireland | ***5-yr:*** *53.1 (42.6-63.1%)e [2000-2004] [79]* | **5-yr:** 67.2 (47.9-84.5%)e [2000-2004] [79] |

DLBCL=diffuse large B-cell lymphoma; F=female; FL=follicular lymphoma; M=male; PBCR=population-based cancer registry.

*****Countries not listed in the table did not provide results for DLBCL or FL. Regional estimates are provided when national estimates were unavailable.

**^†^**The following diagnostic codes defined DLBCL in each country: United States (ICD-0-3 9678, 9679, 9680, 9684); United Kingdom (ICD-0-3 9675, 9678, 9679, 9680, 9684); Australia (ICD-0-3 9680).

**^‡^**Italicized results are for diffuse non-Hodgkin lymphoma. The following diagnostic codes defined diffuse NHL in each country: Australia (ICD-10-83); Brazil (ICD-10-C83); Northern Ireland (ICD-10-C83).

**^§^**The following diagnostic codes defined follicular lymphoma in each country: United States (ICD-0-3 9690, 9691, 9695, 9698); Australia (ICD-10-C82); Brazil (ICD-0-3 9690-91, 9695, 9698); United Kingdom (ICD-0-3 9690, 9691, 9695, 9698); Northern Ireland (ICD-10-C82).

^a^Incidence rates are age-adjusted to the national population.

^b^Incidence rates are age-adjusted to the world standard population.

^c^Incidence rates are crude.

^d^Incidence rates are age-adjusted to the European population.

^e^Survival rates were predicted by period survival analysis.

**Supplementary Table 4. Incidence, Prevalence, and Survival Associated With ALL^*,†^ by Country^‡^**

|  | **Incidence per 100,000**  **(95% CI)**  **[Year(s) of Diagnosis]** | **Prevalence per 100,000 (95% CI)**  **[Date of Estimate]** | **Relative Survival, % (95% CI)**  **[Year(s) of Diagnosis]** |
| --- | --- | --- | --- |
| **North and South America** | |  |  |
| United States | **Total ALL**  All=1.71(1.618–1.804)a; M =1.89 (1.762–2.041)a;  F= 1.53 (1.408–1.658)a  [2007] [18]  **B–cell ALL**  All=1.02 (0.95–1.1)a; M=1.09 (0.98–1.19)a; F=0.96 (0.86–1.06)a  [2007] [18] | **Total ALL**  **1 y:** All= 14.8; M=16.2; F=13.4  **5 y:** All=5.8; M=6.5; F=5.0  **7 y:** All=7.6; M=8.5; F=6.7  [July 1, 2007] [18]  **B–cell ALL**  **1 y:** All = 0.9; M=1.0;F=0.8  **5 y:** All=3.1; M=3.4; F=2.8  **7 y:** All=3.6 M=3.9 F=3.3  [July 1, 2007] [18] | **Total ALL**  **1 y:** All=83.7 (81.4–85.7); M=84.8 (81.8–87.3); F=82.2 (78.8–85.4)  [2006] [18]  **3 y:** All=69.2 (68.1–70.3); M=68.9 (67.4–70.4); F=69.5 (67.8–71.2)  [2001–2006] [18]  **5 y:** All=63.1 (61.1–65.2); M=63.2 (60.4–65.9); F=63.1 (59.5–66.1)  [2000–2001] [18]  **B–cell ALL**  **1 y:** All=83.1 (81.2–84.8); M=84.3 (81.9–86.4); F=81.5 (78.5–84.1)  [2006] [18]  **5 y**: All=65.1 (63.4–66.8); M=64.3 (61.9–66.5); F=66.2 (63.6–68.7)  [2000–2001] [18] |
| Canada | All=1.3 (1.2–1.5)a; M=1.6 (1.4–1.8)a; F=1.1 (0.9–1.2)a  [2007] [43] | Not reported | **5 y:** All=37 (32–42)e,f; M=33 (27–39)e,f;  F=42 (34–50)e,f  [2000–2002] [43] |
| Brazil | *Belo Horizonte [2000][46]: M=3.73b; F=2.35b*  *Cuiaba [2000][46]: M=3.23b; F=0.91b*  *Goiânia [1996–2000][46]: M=2.74b; F=1.71b*  *João Pessoa [1999–2000][46]^:^ M=1.67b; F=1.82b*  *Salvador [1997–2001][46]:M=1.36b; F=1.09b* | Not reported | Not reported |
| **European Union – 5** | |  |  |
| United Kingdom | All=1.2c; M=1.3c; F=1.0c  [2008][47] | Not reported | Not reported |
| England | All=1.1c; M=1.3c; F=1.0c  [2008] [47] | Not reported | Not reported |
| Northern Ireland | All=1.45b; M=1.73b; F=1.19b  [2009] [80]  All=1.24c; M=1.53c; F=0.98c  [2009] [80] | Not reported | **1 y:** 52.2 (41.5–62.9)  **5 y:** 24.1 (14.8–33.3)  [1993–2000] [81] |
| Republic of Ireland | All=1.5b; M=1.7b; F=1.2b All=1.2c; M=1.5c; F=0.98c [2008]^\|\|^ | Not reported | 5 y: 27.3 (15.5–40.5)f [2000–2004] [79] |
| Scotland | All=1.5c; M=1.5c; F=1.5c [2008] [47]   All=2.0 (1.5–2.5)b; M=1.9b; F=2.1b [2008] [82] | **<1 y:** All=0.8; M=1.0; F=0.5 **1–5 y:** All=3.5; M=4.2; F=2.8  **5–10 y:** All=3.4; M=4.0; F=2.9  **10–20 y:** All=6.7; M= 8.0; F=5.6  [Dec. 31, 2007] [55] | Not reported |
| Wales | M=1.6c; F=1.3c  [2009] [83] | **<1 y:** All=1.0d; M=1.30d; F=0.72d  **1–5 y:** All=3.47d; M=4.24d; F=2.74d  **5–10 y:** All=3.44d; M=4.04d; F=2.87d  **10–20 y:** All=5.85d; M= 7.12d; F=4.63d  [Dec. 31, 2008] [84] | **1 y:** All=48.59; M=52.94; F=42.20  [1994–2003]  **5 y:** All=29.97; M=28.34; F=32.45  [1994–2003] [84] |
| Germany | **Hamburg PBCR**  M=0.8b; 0.9c; F=2.1b; 3.0c  [2009] [85]  **Lower Saxony PBCR** M=1.3b; 1.7c ; F=1.4b; 1.7c  [2008] [76]  **Schleswig–Holstein PBCR** M=1.7b; 2.2c; F=1.7b; 2.2c  [2008] [86] | Not reported | Not reported |
| Italy | M=1.5d; F=1.4d  [2005] [87] | **<2 y:** All**=**2; M=2; F=2 **<5 y:** All**=**4; M=4; F=3 **<10 y:** All**=**7; M=9; F=6 **<15 y:** All=10; M=12; F=9 **<20 y:** All=13; M=15; F=11  [Jan. 1, 2006] [88] | ***1 y:*** *All=84: M=86; F=82*  ***3 y:*** *All=73; M=75: F=72*  ***5 y:*** *All=65(63–67); M=66 (64–68);*  *F=63 (63–67)*  *[not reported] [62]* |
| Spain | *Albacete (1998–2002) [89]: M=6.09c; F=4.31c*  *Asturias (1996–2006) [89]: M=5.46c; F=3.46c*  *Pais Vasco (1998–2002) [89]: M=5.13c; F=2.60c*  *Canarias (1997–2002) [89]: M=5.43c; F=3.56c*  *Cuenca (1997–2002) [89]: M=3.51c; F=2.90c*  *Girona (1998–2002) [89]: M=3.99c; F=2.99c*  *Granada (1998–2002) [89]: M=4.88c; F=2.55c*  *Murcia (1997–2002) [89]: M=5.43c; F=4.02c*  *Navarra (1998–2002) [89]: M=4.19c; F=2.26c*  *Tarragona (1998–2002 )[89]: M=4.51c; F=3.25c*  *Zaragoza (1996–2002) [89]: M=5.34c; F=3.45c* | Not reported | Not reported |
| **Asia** |  |  |  |
| China | *1.39d [2006]^¶^* | Not reported | ***Hong Kong:***  ***1 y:*** *All=79.3* ***3 y:*** *All=64.3*  ***5 y:*** *All=60.0; M=60.7; F=58.9 [1996–2001][64]*  ***Qidong:  1 y:*** *All=21.3* ***3 y:*** *All=7.1*  ***5 y:*** *All=4.8; M=3.6; F=9.3*  *[1992–2000][36]* |
| South Korea | All=1.3a; All=1.6b  M=1.5a; M=1.8b  F=1.1a; F=1.4b [2008] [66] | **5 y:** All=4.0a; M=4.4a; F=3.5a  **5 y:**All=4.8b; M=5.3b; F=4.4b  [2008] [66] | **Busan:  1 y:** All=66.0 **3 y:** All=48.0  **5 y:** All=42.2; M=37.7; F=50.1  [1996–2001] [90]  **Incheon:  1 y:** All=67.5 **3 y:** All=50.7  **5 y:** All=50.8; M=49.7; F=51.8  [1997–2001] [91] |
| Japan | M=1.44b; F=1.14b  [not reported] [65] | Not reported | Not reported |
| India | Bangalore: M=1.2b,d; F=0.8b,d  Bhopal: M=0.7b,d; F=0.6b,d  Chennai: M=1.9b,d; F=1.5b,d  Delhi: M=1.8b,d; F=0.9b,d  Mumbai: M=1.2b,d; F=0.9b,d  [2003–2008]^#^ | Not reported | Not reported |
| **Australia** | |  |  |
| Australia | All=1.5a; M=1.8a; F=1.2a;  All=1.7b; M=2.1b; F=1.3b [2005] [70] | Not reported | Not reported |

ALL=acute lymphocytic leukemia; F=female; M=male; PBCR=population-based cancer registry.

*The following diagnostic codes were used for ALL in each country: Australia (ICD-0-3 9272-29, 9835-37); Canada (ICD-0-3 9826, 9835-9837); Italy (ICD-10 C91); United Kingdom (ICD-10 C91.0); Northern Ireland (not reported); Scotland (not reported); England (not reported); Wales (not reported).

^†^Italicized results are for lymphoid leukemia. Rates for lymphoid leukemia were included when results for ALL were unavailable. The following diagnostic codes were used for lymphoid leukemia in each country: Brazil (ICD-10-C91); China (not reported); Germany (ICD-10-C91); India (ICD-10-C91); South Korea (not reported); Spain (not reported).

^‡^Countries not listed in the table did not provide results for ALL or lymphoid leukemia. Regional estimates are provided when national estimates were unavailable.

^§^Survival rates were predicted by period survival analysis.

^||^S. Deady and M. Wagner, personal communication with National Cancer Registry of Ireland, September 24, 2010.

^¶^L. Watson and M. Wagner, personal communication, October 19, 2010.

^#^M. Mitchell and N. Kumar, personal communication, August 8, 2011.

^a^Incidence rates are age-adjusted using the national standard population.

^b^Incidence rates are age-adjusted to world standard population.

^c^Incidence rates are age-adjusted to the European population.

^d^It is unclear which population the rates were adjusted to.

^e^Quebec is excluded from the survival analysis.

^f^Survival results reported for persons 15-99 years of age.

**Supplementary Table 5. Incidence, Prevalence, and Survival Associated With Pediatric ALL^*,†^ by Country^‡^**

|  | **Incidence per 1,000,000 (95% CI)**  **[Year(s) of Diagnosis]** | **Prevalence per 100,000 (95% CI)**  **[Date of Estimate]** | **Relative Survival, % (95% CI)**  **[Year(s) of Diagnosis]** |
| --- | --- | --- | --- |
| **North and South America** | |  |  |
| United States | **Total ALL**  All=35.14 (30.51–40.27)a; M=37.49 (30.89–45.08)a; F=32.66 (26.39–39.98)a  **0 y:** 22.13 (10.12–42.02)  **1–4 y:** 69.82 (57.39–84.16)  **5–9 y:** 26.52 (19.69–34.97)  **10–14 y:** 19.88 (14.06–27.28)  [2007] [18]  **B–cell pediatric ALL**  All= 1.77 (1.70–1.84)a; M=1.86 (1.76–1.96)a; F=1.67 (1.58–1.77)a  **0 y:** 0 (0–0.907)  [2007] [18] | **1 y:** 3.3  **5 y:** 6.0  **7 y:** 21.1  [July 1, 2007] [18] | **5 y:** 87.23 (85.6–88.7)  [2007] [18] |
| Canada | *All=34.0a,f*  *[2003–2007] [92]* | Not reported | ***5 y:*** *89 (87–91)g*  *[2002–2006] [92]* |
| Brazil | Belem [1997–2001][93]: M=24.2b; F=26.6b  Manaus [1999–2002] [93]: M=60.1b; F=53.0b  Aracaju [1998–2002] [93]: M=28.3b; F=14.9b  Fortaleza [1998–2002] [93]: M=27.6b; F=26.2b  Recife [1997–2001][93]: M=45.7b; F=45.5b  João Pessoa [2000–2004] [93]: M=27.0b; F=17.2b  Natal [1998–2001] [93]: M=44.6b; F=27.0b  Salvador [1998–2002] [93]:M=22.7b; F=12.8b  Cuiaba [2000–2004][93]:M=58.1b; F=41.5b  Brasilia [1999–2002] [93]: M=32.1b; F=29.0b  Goiania [1999–2003] [93]: M=61.3b; F=44.8b  Belo Horizonte [2000–2003] [93]:M=37.5b; F=30.3b  Sao Paulo [1998–2002] [93]:M=38.8b; F=30.3b  Jau [2000–2004] [93]:M=35.3b; F=69.0b  Curitiba [1999–2003] [93]:M=39.8b; F=62.0b  Porto Alegre [1999–2003] [93]:M=56.1b; F=28.1b | Not reported | Not reported |
| **European Union –5** | |  |  |
| United Kingdom | All=36.11b  **0 y:** 17.95  **0–4 y:** 66.68  **5–9 y:** 28.32  **10–14 y:** 17.01  [1991–2000] [94] | Not reported | **5 y:** 88  [2001–2005] [94] |
| Republic of Ireland | All=33.7b; M=37.0b; F=30.2b All=33.4b; M=36.5b; F=30.3b [2008]^§^ | Not reported | Not reported |
| Wales | All=34.4b  **0 y:** 17.7  **0–4 y:** 58.6  **5–9 y:** 29.2  **10–14 y:** 18.9  [1991–2005] [94] | Not reported | Not reported |
| France | *All=35.7b*  ***0 y:*** *14.3*  ***1–4 y:*** *61.4*  ***5–9 y:*** *31.2*  ***10–14 y:*** *19.0*  *[2000–2004] [19]* | Not reported | **5 y**: All=82 (80–83); M=81 (79–82); F=83 (81–85)  [2000–2004] [95] |
| Germany | *All=43.6b; M=46.5b; F=40.5b*  ***0 y:*** *17.5*  ***1–4 y:*** *79.9*  ***5–9 y:*** *34.8*  ***10–14 y:*** *21.7*  *[2000–2009] [21]* | Not reported | ***5 y:*** *All=90*  ***10 y:*** *All=88*  ***15 y:*** *All=87*  *[1980–2007] [21]* |
| Italy | All= 43.0 (39.4–46.7)a; 46.2 (39.1–53.3)c  M=47.0 (41.6–52.3)a; F=38.9 (33.9–43.9)a  [1998–2002] [62] | Not reported | **Cumulative survival:**  **3 y:** 88.9 (86.2–91.6)  **5 y:** 86.2 (83.0–89.4)  [1998–2002] [62] |
| Spain | *35.2b [1983–2002] [20]* | Not reported | **5 y:** 76.1i  [1991–2002] [20] |
| **Asia** |  |  |  |
| China | ***Shanghai*** *M=31.4b; F=33.5b*  *[2003–2005][96]* ***Guangzhou*** *All=43.9e*  *[2000–2004] [97]* | Not reported | Not reported |
| South Korea | *All=28.7a; M=30.1a; F=27.1a*  *All=30.3b; M=31.5b; F=29.0b*  ***0–4 y:*** *All=47.3; M=46.2; F=48.6*  ***5–9 y:*** *All=21.9; M=26.6; F=16.7*  ***10–14 y:*** *All=17.1; M=17.5; F=16.5*  *[not reported] [66]* | ***0–4 y:*** *All=7.8; M=7.6; F=7.9*  ***5–9 y:*** *All=15.7; M=16.9; F=14.3*  ***10–14 y:*** *All=6.7; M=7.1; F=6.2*  *[2008] [66]* | Not reported |
| India^b^ | *Ahmedabad: M=9.7b; F=13.7b*  *Bangalore M=27.9b; F=18.5b*  *Barshi M=17.9b; F=13.5b*  *Bhopa M=11.8b; F=14.6b*  *Chennai M=47.1b; F=28.6b*  *Delhi M=38.4b; F=14.7b*  *Mumbai M=21.5b; F=17.1b*  *North East M=11.3b; F=8.5b*  *[2006] [98]* | Not reported | *Chennai* ***5 y:*** *39j*  *[1990–2001] [98]* |
| **Australia** |  |  |  |
| Australia | *All=40.8 (38.8 – 42.8)b*  *[1997–2006] [31]* | Not reported | **5 y:** All=85.0 (83–87)k;M=82.9 (80–85)k; F=87.5 (85–90)k  [1983–2006]^\|\|^ |

F=female; M=male; pediatric B-cell acute lymphoblastic leukemia (ALL).

*The following diagnostic codes were used to define pediatric ALL: Northern Ireland (not reported); Italy (not reported); United States (ICD-0-3 9826, 9835-37).

^†^Italicized results are for pediatric lymphoid leukemia. The following diagnostic codes were used to define pediatric lymphoid leukemia: Canada (not reported); Brazil (ICCC-3 Ia); UK (not reported); France (ICCC-2 Ia); Germany (ICCC-3 Ia); Italy (not reported); Spain (not reported); China (ICD-9 204); South Korea (not reported); India (ICD-10 C91); Australia (ICCC-3 Ia).

^‡^Countries not listed in the table did not provide results for pediatric ALL or pediatric lymphoid leukemia. Regional estimates are provided when national estimates were unavailable.

^§^S. Deady and M. Wagner, personal communication with National Cancer Registry of Ireland, September 24, 2010.

^||^J. Aitken and M. Mitchell, personal communication, February 13, 2011.

^a^Incidence rates are age-adjusted using the national population.

^b^Incidence rates are age adjusted to the world standard population.

^c^Incidence rates are age-adjusted to the European population.

^d^It is unclear which population the rates were adjusted to.

^e^Incidence rates are crude.

^f^Incidence rates, 0-19 years of age

^g^Survival rates are observed survival, 0-19 years of age

^h^Excludes Ireland.

^i^It is unclear whether this is relative or cumulative survival.

^j^Survival rates are observed survival.

^k^Survival rates were predicted by period survival analysis.

**Supplementary Table 6. Incidence, Prevalence, and Survival Associated With MM^*^ by Country^†^**

|  | **Incidence per 100,000**  **(95% CI) [year(s) of diagnosis]** | **Prevalence per 100,000 (95% CI)**  **[date of estimate]** | **Relative Survival (%) (95% CI)**  **[year(s) of diagnosis]** |
| --- | --- | --- | --- |
| **North and South America** | | | |
| United States | All=5.60 (5.44–5.78)a; M=7.31 (7.02–7.61)a; F=4.30 (4.10 – 4.50)a  [2007] [18] | **1 y:** All=3.7; M=4.1; F=3.4  **5 y:** All=12.8; M=14.0; F=11.5  **7 y:** All=14.9; M=16.4; F=13.6  [July 1, 2007] [18] | **1 y:** All: 73.84; M: 74.51; F: 73.06  [2006] [18]  **3 y:** All: 53.13; M: 53.92; F: 52.22  [2001–2006] [18]  **5 y:** All: 34.95; M: 35.90; F: 33.90  [2000–2001] [18] |
| Canada | All=4.8 (4.5–5.0)a; M=5.7 (5.4–6.1)a; F=4.0 (3.7–4.3)a  [2007] [43] | **2 y:** All=4.7b; M= 5.5b; F= 3.9b  **5 y:** ALL=8.4b; M= 9.9b; F=7.1b  **10 y:** ALL=10.9b; M= 12.8b; F= 9.2b  [Jan. 1, 2005] [44] | **1 y:** 73 (71–74)  **3 y:** 50 (49–52)  **5 y:** 37 (35–38)  **10 y:** 22 (20–24)  [2004–2006] [45]^‡, §. #^ |
| Brazil | Belo Horizonte (2000) [46]: M=3.84b; F=3.54b  Cuiaba (2000) [46]: M=1.66b; F=2.78b  Goiânia (1996–2000) [46]: M=2.01b; F=1.64b  João Pessoa (1999–2000) [46]: M=2.39b; F=3.88b  Salvador (1997–2001) [46]: M=1.42b; F=1.31b | Not reported | Not reported |
| **European Union–5** | | | |
| United Kingdom | All=5.3c; M=6.5c; F=4.3c  [2008] [47] | **1 y:** All=3.7(3.5–3.8)c; M=4.5 (4.3–4.7)c; F=3.0 (2.8–3.1)c  **5 y:** All=12.1 (11.8–12.4)c; M=14.9 (14.5–15.3)c;  F=9.7 (9.4–10.0)c  **10 y:** All=16.0 (15.7–16.3)c;  M=19.7 (19.2–20.1)c; F=12.9 (12.5–13.3)c  [Dec. 31, 2006] [48] | **1 y:** M=67 (59–73) ^‡^; F=65 (57–71) ^‡^  **5 y:** M=27 (24–31) ^‡^; F=26 (23–30) ^‡^  **10 y:** M=15 (13–18) ^‡^; F=13 (10–15)  [2000–2001] [99]^‡^ |
| England | All=5.4c; M=6.6c; F=4.4  [2008] [47] | **1 y:** All=3.6 (3.4–3.7)c; M=4.4 (4.1–4.6)c; F=2.9 (2.7–3.1)c  **5 y:** All=11.9 (11.6–12.1)c: M=14.5 (14.1–15.0)c: F=9.6 (9.3–10.0)c  **10 y:** All=15.7 (15.4–16.1)c; M=19.2 (18.7–19.7)c: F=12.8 (12.4–13.2)c  **20 y:** All=17.5 (17.2–17.8)c: M=21.4 (20.9–22.0)c: 14.2 (13.8 – 14.6)c  [Dec. 31, 2006] [48] | **1 y:** M=68.7 (67.7–69.7)d,^#^; F=70.8 (69.8–71.9)d,^#^  **5 y:** M=35.6 (34.0–37.1)d,^#^; F=35.1 (33.4–36.8)d,^#^  [2004–2008] [50] |
| Ireland | All=5.5c; M=7.1c; F=4.1c  [2000–2004] [51] | Not reported | **1 y:** All=68.8 (66.4–71.3); M=67.5 (64.3–70.8); F=70.8 (67.3–74.5)  **5 y:** All=34.9 (32.1–38.1); M=32.9 (29.1–37.2); F=38.5 (34.5–43.0)  [2000–2004] [51]^‡^ |
| Republic of Ireland | M=5.0b; F=2.8b M=3.3c; F=1.8c [2009] [100] | Not reported | **5 y:** 42.1^#^  [2003–2007] [100] |
| Northern Ireland | All=4.9c; M=6.7c; F=3.5c  [2008] [47] | **1 y:** All=5.5 (4.5–6.6)c; M=6.3 (4.6–8.0)c; F=4.8 (3.5–6.1)c  **5 y:** All=16.0 (14.2–17.8)c; M=19.7 (16.8–22.7)c; F=13.0 (10.8–15.2)c  **10 y:** All=21.3 (19.2–23.4)c; M=26.0 (22.6–29.4)c; F=17.3 (14.8–19.9)c  [Dec. 31, 2006] [48] | **1 y:** All=70.8 (66.8–75.0); M=69.9 (64.5–75.6); F=72.4 (66.7–78.6)  **5 y**: All=39.8 (34.9–45.4); M=36.7 (30.0–45.0)F=45.1 (38.8–52.5)  [2000–2004] [51]^‡^ |
| Scotland | All=5.3c; M=6.5c; F=4.3c  [2008] [47]  All=3.5 (3.1–3.9)b; M=4.3 (3.7–4.9)b; F=2.8 (2.3–3.2)b [2008] [55] | **Prevalence rate (proportion [%])**  **<1 y:** All=5.4 (0.005); M=5.7; (0.006); F=5.1 (0.005)  **1–5 ys:** All=11.6 (0.012); M=13.4 (0.013); F=9.9 (0.010)  **5–10 ys:** All=4.9 (0.005); M=5.8 (0.002); F=4.1 (0.004)  **10–20 ys:** All= 2.0 (0.002); M=2.0 (0.002); F=2.0 (0.002)  [Dec. 31, 2007] [101] | **1 y:** All=72.9; M=72.9; F=73.0  **3 y:** All=50.2; M=51.8;  F=48.5  **5 y:** All=37.3; M=39.4  F=35.1  [2003–2007] [101] |
| Wales | M=8.1c; F=4.6c  [2009] [83] | **1 y:** All=4.1 (3.5–4.7)c; M=5.1 (4.1–6.2)c; F=3.3 (2.5–4.0)c  **5 y:** All=13.4 (12.3–14.6)c; M=17.3 (15.4–19.2)c;  F=10.0 (8.7–11.4)c  **10 y:** All=17.8 (16.4–19.1)c; M=22.7 (20.5–24.9)c; F=13.4 (11.9–15.0)  [Dec. 31, 2006] [48] | **1 y:** All=61.89; M=64.39; F=59.17  **3 y:** All=38.60; M=38.62; F=38.47  **5 y:** All=27.18; M=28.45; F=25.74  [1992–2001] [102] |
| France | M=4.6b; F=2.9b  [2005][22]  M=7.1c; F=4.5c  [2008] [26] | **5 y**: M=16.4c; F=11.5c  [Dec. 31, 2002] [39] | Not reported |
| Germany | M=5.2c; F=3.5c  [2008] [26] | Not reported | **5 y:** 41.1  [2002–2006] [40]^‡^ |
| Italy | M=6.4c; F=5.5c  [2008] [26] | **<2 y:** All**=**13; M=13; F=12 **<5 y:** All**=**24; M=26; F=22 **<10 y:** All**=**33; M=35; F=31 **<15 y:** All=37; M=39; F=36 **<20 y:** All=39; M=41; F=37  [Jan. 1, 2006] [61] | **1 y:** All=77; M=77; F=77  **3 y:** All=55; M=55; F=55  **5 y:** All=42 (41–44); M=43 (41–46); F=41 (39–43)  [not reported] [62] |
| Spain | M=5.0c; F=4.3c  [2008] [26] | Not reported | Not reported |
| **Asia** | | | |
| China | All=1.24d,e  [2006]^\|\|^  M=0.6b,e; F=0.3b,e  [2005][25] | Not reported | **Hong Kong:**  **1 y:** All=65.1 **3 y:** All=42.4  **5 y:** All=31.0; M=31.7; F=30.4 [1996–2001] [64] **Qidong:  1 y:** All=15.1 **3 y:** All=10.1  **5 y:** All=11.1; M=14.1; F=8.4  [1992–2000] [36] |
| Japan | All=2.0a; M=2.4a; F=1.7a  All=1.4b; M=1.6b; F=1.2b  [2006] [103] | Not reported | Not reported |
| South Korea | All=1.4a; All=1.4b  M=1.7a; M=1.6b  F=1.2a; F=1.2b [2008] [66] | **5 y:** All=3.2a; M=3.6a; F=2.8a **5 y:**All=3.1b; M=3.5b; F=2.8b  [2008] [66] | **5 y:** All=33.2; M=33.7; F=32.8  [2004–2008] [66] |
| India | Silchar Town: M=0.4b; F=not reportedb  Imphal West District: M=0.6b; F=1.2b  Kamrup Urban District: M=1.4b; F=1.0b  Aizawl District: M=not reported; F=0.8b  Mizoram State: M=0.3b; F=0.3b  Dibrugarh District: M=0.2b; F=1.1b  Mizoram State, Excl. Aizawl: M=0.4b; F=not reported  Sikkim State: M=0.4b; F=0.4b  [2005–2006] [104] | Not reported | Not reported |
| **Australia** | | | |
| Australia | All=5.6a; M=7.0a; F=4.4a  All=3.9b; M= 4.8b; F=3.1b  [2007][71] | Not reported | Not reported |

F=female; M=male; MM=multiple myeloma.

*The following diagnostic codes were used to define multiple myeloma: United States (ICD-O-3 9732, 973); Canada (ICD-O-3 9731, 9732, and 9734); Brazil (ICD-10 C90); England (ICD-10 C90); Northern Ireland (ICD-10 C90); Scotland (IC-10 C90); Wales (ICD-10 C90); France (ICD-10 C88-90); Germany (ICD-10 C88-90); Italy (ICD-10 C88-90); Spain (ICD-10 C88-90); China (ICD-10 C88-90); Japan (ICD-10 C88-C90); South Korea (not reported); India (ICD-10 C90); Australia (ICD-10 C90).

^†^Countries not listed in the table did not provide results for MM. Regional estimates are provided when national estimates were unavailable.

^‡^Survival rates were predicted using period survival analysis.

^§^Quebec is excluded from the survival analyses.

^#^Survival results reported for persons 15-99 years of age.

^||^L. Watson and M. Wagner, personal communication, October 19, 2010.

^a^Age-adjusted using the national population.

^b^Age-adjusted to the world standard population.

^c^Age-adjusted to the European population.

^d^It is unclear which population the gender-specific rates were adjusted to.

^e^Incidence rates are estimated.

**Supplementary Table 7. Incidence, Prevalence, and Survival Associated With CLL* by Country^†^**

|  | **Incidence per 100,000**  **(95% CI)**  **[Year(s) of Diagnosis]** | **Prevalence per 100,000 (95% CI)**  **[Date of Estimate]** | **Relative Survival, % (95% CI)**  **[Year(s) of Diagnosis]** |
| --- | --- | --- | --- |
| **North and South America** | |  |  |
| United States | All=5.0 (4.9–5.2)a, M=6.9 (6.6–7.2)a; F=3.6 (3.4–3.8)a  [2007] [18] | **1 y:** All=0; M=0; F=0  **5 y:** All=15; M=20; F=10  **7 y:** All=20; M=30; F=20  [July 1, 2007] [18] | **1 y:** All=91.6 (90.6–92.6); M=91.8 (90.4–93.0); F=91.5 (89.8–92.8)  [2006] [18]  **3 y:** All=85.1 (84.4–85.9); M=84.4 (83.4–85.3); F=86.2 (85.1–87.3)  [2001–2006][18]  **5 y:** All=77.7 (76.5–78.9); M=75.9 (74.3–77.5); F=80.3 (78.3–82.1)  [2000–2001] [18] |
| Canada | All=5.0 (4.8–5.2)a; M=6.8 (6.4–7.1)a; F=3.6(3.3–3.8)a  [2007] [43] | Not reported | **5 y:** All=75 (73–77); M=73 (70–76); F=79 (76–82)  [2000–2002] [105]^§#^ |
| Brazil | *Belo Horizonte [2000] [46]: M=3.73b; F=2.35b*  *Cuiaba [2000][46]: M=3.23b; F=0.91b*  *Goiânia [1996–2000] [46]: M=2.74b; F=1.71b*  *João Pessoa [1999–2000] [46]^:^ M=1.67b; F=1.82b*  *Salvador [1997–2001] [46]:M=1.36b; F=1.09b* | Not reported | Not reported |
| **European Union – 5** | |  |  |
| United Kingdom | All=3.3c; M=4.5c; F=2.2c  [2008] [47] | Not reported | All=73.0 (70.9–75.1) [2000–2002][23] |
| England | All=3.4c; M=4.6c; F=2.3c  [2008] [47] | Not reported | Not reported |
| Northern Ireland | All=2.9c; M=4.9c; F=1.5c  [2008] [47] | Not reported | **1 y:** 86.4 (82.2–90.6)  **5 y:** 59.2 (52.5–65.9)  [1993–2000] [81] |
| Republic of Ireland | All=3.1b; M=4.3b; F=2.1b All=4.7c; M=6.6c; F=3.2c [2008]^\|\|^ | Not reported | **5 y:** 72.2 (66.9–77.1)f [2000–2004] [79] |
| Scotland | All=2.7c; M=3.8c; F=1.8c  [2008] [47] All=1.9 (1.6–2.1)b; M=2.5 (2.0–3.0)b; F=1.2 (0.9–1.5)b [2008] [55] | **<1 y:** All=3.4; M=4.5; F=2.4 **1–5 y:** All=13.7; M=17.1; F=10.5  **5–10 y:** All=12.1; M=14.6; F=9.8  **10–20 y:** All=7.6; M=8.7; F=6.6  [Dec. 31, 2007] [55] | Not reported |
| Wales | M=7.1c; F=2.7c  [2009] [83] | **<1 y:** All=3.67d; M=4.45d; F=2.94d  **1–5 y:** All=14.63d; M=17.04d; F=12.34d  **5–10 y:** All=10.49d; M=12.59d; F=8.49d  **10–20 y:** All=7.45d; M=8.28d; F=6.66d  [Dec. 31, 2008] [84] | **1 y:**All=82.63; M=82.99; F=82.08  **5 y:** All=65.01; M=62.86; F=68.41  [1994–2003] [84] |
| France | M=3.6b; F=2.0b [2005][22] | Not reported | Not reported |
| Germany | **Hamburg PBCR**  M=3.6b; 2.3c F=2.0b; 1.3c  [2009][85]  **Lower Saxony PBCR** M=4.3b; 2.9c F=2.3b; 1.6c  [2008] [76]  **Schleswig–Holstein PBCR** M=4.7b; 3.0c F=2.3b; 1.5c  [2008] [86] | Not reported | Not reported |
| Italy | M=3.5d; F=1.6d  [2005] [87] | **<2 y:** All**=**7; M=8; F=6 **<5 y:** All**=**16; M=20; F=13 **<10 y:** All**=**28; M=31; F=23 **<15 y:** All=33; M=37; F=29 **<20 y:** All=35; M=40; F=31  [Jan. 1, 2006] [61] | ***1 y:*** *All=84: M=86; F=82*  ***3 y:*** *All=73; M=75: F=72*  ***5 y:*** *All=65(63–67); M=66 (64–68); F=63 (63–67)*  *[not reported] [62]* |
| Spain | *Albacete (1998–2002) [89]: M=6.09c; F=4.31c*  *Asturias (1996–2006) [89]: M=5.46c; F=3.46c*  *Pais Vasco (1998–2002) [89]: M=5.13c; F=2.60c*  *Canarias (1997–2002) [89]: M=5.43c; F=3.56c*  *Cuenca (1997–2002) [89]: M=3.51c; F=2.90c*  *Girona (1998–2002 )[89]: M=3.99c; F=2.99c*  *Granada (1998–2002) [89]: M=4.88c; F=2.55c*  *Murcia (1997–2002) [89]: M=5.43c; F=4.02c*  *Navarra (1998–2002) [89]: M=4.19c; F=2.26c*  *Tarragona (1998–2002) [89]: M=4.51c; F=3.25c*  *Zaragoza (1996–2002) [89]: M=5.34c; F=3.45c* | Not reported | Not reported |
| **Asia** |  |  |  |
| China | *1.39d [2006]^¶^* | Not reported | ***Hong Kong:***  ***1 y:*** *All=79.3* ***3 y:*** *All=64.3*  ***5 y:*** *All=60.0; M=60.7; F=58.9 [1996–2001] [64]*  ***Qidong:  1 y:*** *All=21.3* ***3 y:*** *All=7.1*  ***5 y:*** *All=4.8; M=3.6; F=9.3*  *[1992–2000] [36]* |
| South Korea | *All=1.3a; All=1.6b*  *M=1.5a; M=1.8b*  *F=1.1a; F=1.4b*  *[2008] [66]* | ***5 y:*** *All=4.0a; M=4.4a; F=3.5a*  ***5 y:****All=4.8b; M=5.3b; F=4.4b*  *[2008] [66]* | ***Busan:  1 y:*** *All=66.0* ***3 y:*** *All=48.0*  ***5 y:*** *All=42.2; M=37.7; F=50.1*  *[1996–2001] [90]*  ***Incheon:  1 y:*** *All=67.5* ***3 y:*** *All=50.7*  ***5 y:*** *All=50.8; M=49.7; F=51.8*  *[1997–2001] [91]* |
| India | Bangalore: M=0.4g; F=0.3g  Bhopal: M=0.6g; F=0.2g  Chennai: M=0.2g; F=0.1g  Delhi: M=0.7g; F=0.3g  Mumbai: M=0.3g; F=0.2g  [2003–2008]^#^ | Not reported | Not reported |
| **Australia** |  |  |  |
| Australia | All=4.9 (4.6–5.2)a; M=6.6 (6.1–7.1)a, F=3.5 (3.2–3.9)a [2007][78]  All=3.0b; M=4.0b; F=2.1b  [2007] [71] | Not reported | Not reported |

CLL=chronic lymphocytic leukemia; F=female; M=male; PBCR=population-based cancer registry.

*The following diagnostic codes were used to define CLL: Australia (ICD-0-3 9670 and 9823); United Kingdom (ICD-10 C911); England (ICD-10 C911); Northern Ireland (ICD-10 C911); Scotland (ICD-10 C911); Wales (ICD-10 C911); France (ICD-0-3 98233); Italy (ICD-1- C91.1). Italicized results are for lymphoid leukemia. Rates for lymphoid leukemia were included when results for ALL were unavailable. The following diagnostic codes were used for lymphoid leukemia in each country: Brazil (ICD-10-C91); China (not reported); Germany (ICD-10-C91); India (ICD-10-C91); South Korea (not reported); Spain (not reported).

^†^Countries not listed in the table did not provide results for CLL or lymphoid leukemia. Regional estimates are provided when national estimates were unavailable.

^§^Quebec is excluded from the survival analyses.

^#^Survival results reported for persons 15-99 yrs of age.

^||^S. Deady and M. Wagner, personal communication with National Cancer Registry of Ireland, September 24, 2010.

^¶^L. Watson and M. Wagner, personal communication, October 19, 2010.

^#^M. Mitchell and N. Kumar, personal communication, August 8, 2011.

^a^Incidence rates are age-adjusted using national standard population.

^b^Incidence rates are age adjusted to the world standard population.

^c^ Incidence rates are age-adjusted to the European population.

^d^It is unclear which population the rates were adjusted to.

^e^Incidence rates are crude.

^f^Survival rates were predicted by period survival analysis.

^g^Age adjustment method is unclear.
